# Peptide-reinforced, photocrosslinkable PEG-based hydrogels

**DOI:** 10.1039/d5lp00335k

**Published:** 2026-01-05

**Authors:** Sam Russell, Daseul Jang, Jessica Thomas, Patrick Grysan, Linus Sprandl, Markus Biesalski, LaShanda T. J. Korley, Nico Bruns

**Affiliations:** a Department of Chemistry, Technical University of Darmstadt Peter-Grünberg-Straße 4 64287 Darmstadt Germany nico.bruns@tu-darmstadt.de; b Centre for Synthetic Biology, Technical University of Darmstadt Peter-Grünberg-Straße 4 64287 Darmstadt Germany; c Department of Pure and Applied Chemistry, University of Strathclyde Thomas Graham Building 295 Cathedral Street Glasgow G1 1XL UK; d Materials Research and Technology, Luxembourg Institute of Science and Technology 5 Avenue des Hauts-Fourneaux Esch-sur-Alzette L-4362 Luxembourg; e Department of Materials Science and Engineering, University of Delaware 127 The Green 209 DuPont Hall Newark DE 19716 USA; f Department of Chemical and Biomolecular Engineering, University of Delaware 150 Academy Street Newark DE 19716 USA

## Abstract

Hydrogels are polymer networks that swell in aqueous solvents. These materials have applications in many fields, including drug delivery, tissue engineering, and soft robotics. For example, polyethylene glycol (PEG) diacrylate is often used as a light-curable crosslinker for the synthesis of PEG-based hydrogels, *e.g.*, in bioinks for 3D printing. However, a common limitation of PEG hydrogels is their typically poor mechanical properties, particularly when in a swollen state. The mechanical strength of natural polymeric materials, such as spider silk and collagen, arises from the formation of hierarchical secondary protein structures that unfold under mechanical load. Here, we present a bio-inspired approach to reinforcing PEG-based hydrogels that mimics these hierarchical structures by incorporating poly(β-benzyl-l-aspartate) (PBLA) blocks between cross-linking end groups and PEG chain segments. We used this peptide-containing crosslinker in combination with a small hydrophilic comonomer, 2-hydroxyethyl acrylate, to synthesise PHEA-*linked by*-(PBLA-*b*-PEG-*b*-PBLA) co-networks with tailored compositions, yielding improved and tailorable mechanical properties. This approach affords hydrogels with increased strength and toughness while retaining the networks’ swelling ability. This research presents a promising avenue for developing robust photocrosslinkable hydrogels with broad practical applications.

## Abstract 2

Peptide blocks close to the crosslinking points mechanically reinforce photocurable PEG-based hydrogels. These polymer conetworks could be applied as biomaterials or for controlled drug delivery systems.

## Graphical abstract

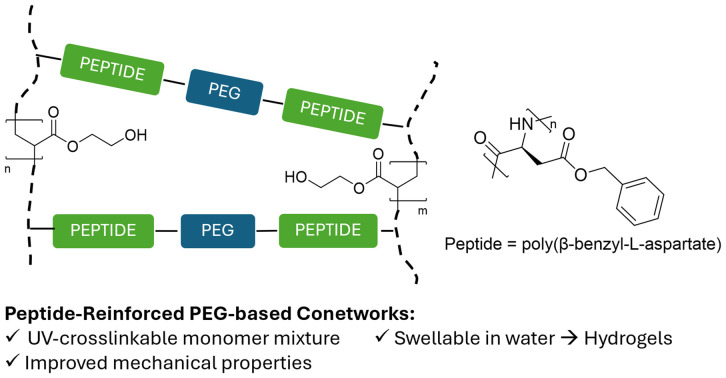

## 1. Introduction

Hydrogels are three-dimensional, cross-linked, hydrophilic polymer networks swollen in water due to the presence of hydrophilic chain segments within their network structure. These materials have applications in many fields, including drug delivery,^1^ tissue engineering,^2^ and soft robotics.^3^ Polyethylene glycol (PEG)-based hydrogels are commonly used in biomedical applications due to their high biocompatibility, serving as vehicles for drug delivery,^4–6^ or as scaffolds and matrices for tissue engineering and cell transplantation.^7^ Moreover, many bioinks for 3D printing of cells and cell scaffolds are based on light-curable PEG-dimethacrylate and PEG-diacrylate crosslinkers.^8,9^ However, a common limitation of synthetic hydrogels is their typically poor mechanical properties, particularly when in a swollen state, which limits their use in load-bearing or high-stress environments.^10^ Numerous examples in the literature demonstrate methods for enhancing the mechanical strength of hydrogels, including the incorporation of reinforcing nanoparticles,^11^ double networks,^12,13^ ionic crosslinking,^14^ force-triggered reactions,^15^ and entanglement of the polymer chains.^16^ There has also been extensive research into improving the mechanical properties, specifically of PEG-based hydrogels, including the incorporation of slide-ring motives,^17^ or bimodal “all-PEG” networks making use of PEG macromonomers and four-arm tetra-PEG macrophotoinitiators.^18^ Most of these approaches utilise a secondary mechanism to dissipate the energy within the material. While each improves specific aspects of hydrogel mechanics, many reinforcement strategies suffer from trade-offs, such as increased stiffness at the expense of extensibility or limited long-term stability due to reversible bonding mechanisms. As a result, researchers continue to explore novel design strategies, including biomimetic approaches, to achieve robust yet flexible hydrogel networks.

The mechanical robustness of natural polymeric materials, such as spider silk and collagen, arises from the formation of hierarchical secondary protein structures that unfold under mechanical load.^19^ Korley and coworkers have taken inspiration from these hierarchical structures and employed peptides into polyurethanes to improve their mechanical properties, creating ABA block copolymers with PEG or polydimethylsiloxane (PDMS) as B blocks and peptidic A blocks comprising poly(β-benzyl-l-aspartate) (PBLA), poly(γ-benzyl-l-glutamate) (PBLG), or poly(ε-carbobenzyloxy-l-lysine) (PZLY).^20–23^ The formation of β-sheets and α-helices by the peptides within the structures reinforces the materials, and the mechanical properties can be tailored by the length of the blocks.^24^ In prior work, we further developed this concept by incorporating peptide moieties into amphiphilic polymer conetworks (APCNs).^25,26^ To this end, we synthesised methacrylate-functionalised ABA peptidic triblock copolymers to use as crosslinkers in poly(2-hydroxyethyl acrylate)-*linked by*-polydimethylsiloxane (PHEA-*l*-PDMS) APCNs, demonstrating that the reinforcement with peptides was possible and an effective way to improve these networks. In analogy to APCNs,^27–29^ fully hydrophilic polymer conetworks, *i.e.*, hydrogels when swollen in water, can be synthesised by radical copolymerisation of 2-hydroxyethyl acrylate (HEA) or 2-hydroxyethyl methacrylate (HEMA) with an α,ω-methacrylate-functionalised PEG macromolecular crosslinker. The resulting PHEA-*l*-PEG conetworks have been used as electrolytes for lithium-ion batteries^30^ and as a stationary phase for protein separation.^31^ PHEMA-*l*-PEG networks have been reported as a support for lipid bilayers on electrodes,^32^ and for drug delivery.^33^ Here, we present a bio-inspired approach to reinforce these hydrogels by incorporating poly(β-benzyl-l-aspartate) (PBLA) blocks between cross-linking points and PEG chain segments, yielding PHEA-*l*-(PBLA-*b*-PEG-*b*-PBLA) networks. This approach enables hydrogels to exhibit increased strength and toughness while retaining their ability to swell. Thus, this research presents a promising avenue for developing robust hydrogels with broad practical applications.

## 2. Results and discussion

### 2.1 Synthesis of peptidic and non-peptidic polymer conetworks

**Fig. 1:**
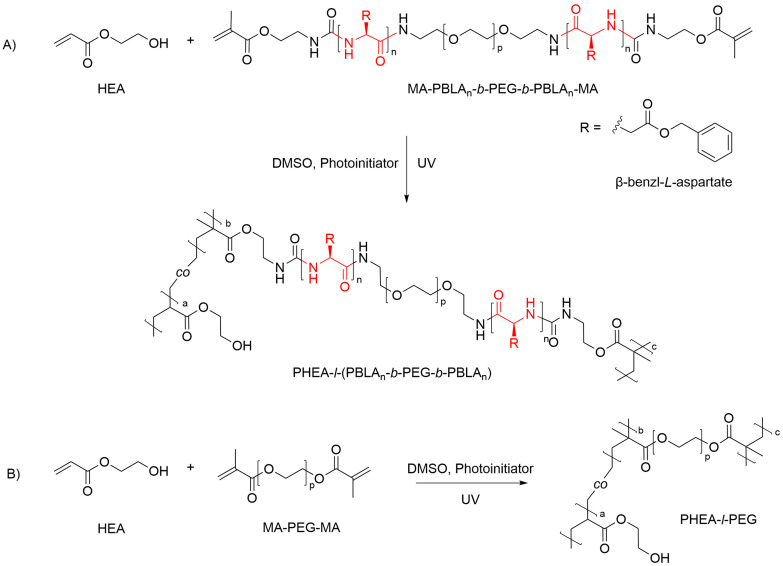
Reaction schemes depicting the synthesis of the conetworks. (A) Synthesis of PHEA-*l*-(PBLA-*b*-PEG-*b*-PBLA). (B) Synthesis of PHEA-*l*-PEG.

The PBLA-*b*-PEG-*b*-PBLA triblock copolymers were prepared by ring-opening polymerisation of β-benzyl-l-aspartate *N*-carboxyanhydride (BLA–NCA) with a PEG macroinitiator with NH_2_ end groups (4000 g mol^−1^) according to an established procedure.^34^ To use the peptide triblock copolymer as a crosslinker for the networks, methacrylate endgroups were installed on either side by reaction with isocyanatoethyl methacrylate to yield α,ω-methacrylate-functionalised MA-PBLA-*b*-PEG-*b*-PBLA-MA. The characterisation data of the block copolymers can be found in the SI (Fig. S1–S3). For comparison, a commercially available, α,ω-methacrylate-functionalised PEG (4000 g mol^−1^) was used to synthesise non-peptidic PHEA-*l*-PEG networks. Polymer network films were prepared with a thickness of 150–200 µm by UV-initiated radical copolymerisation of HEA with either MA-PBLA-*b*-PEG-*b*-PBLA-MA or MA-PEG-MA as crosslinker, using dimethyl sulfoxide (DMSO) as the solvent and Irgacure 2959 as the photoinitiator (Fig. 1). Different hydrogel compositions were prepared with 30, 50, and 70 wt% HEA in the reaction mixture, and with varying degrees of polymerisation of both peptide block (*n*) of the crosslinker. To accommodate spacing limitations in the figure captions and text, PHEA-*l*-(PBLA_20_-*b*-PEG-*b*-PBLA_20_) PHEA-*l*-(PBLA_25_-*b*-PEG-*b*-PBLA_25_) are shortened to PHEA-*l*-PBLA_20/25_, followed by the composition as YY : ZZ, where YY is PHEA wt%, and ZZ is the crosslinker wt%. For example, PHEA-*l*-PBLA_20_ 50 : 50 describes a sample with 50 wt% HEA and 50% crosslinker and 20 repeat units of BLA in each peptide block.

### 2.2 Secondary structure and content of the conetworks

#### 2.2.1 Attenuated total reflectance-Fourier transform infrared (ATR-FTIR) spectroscopy

**Fig. 2:**
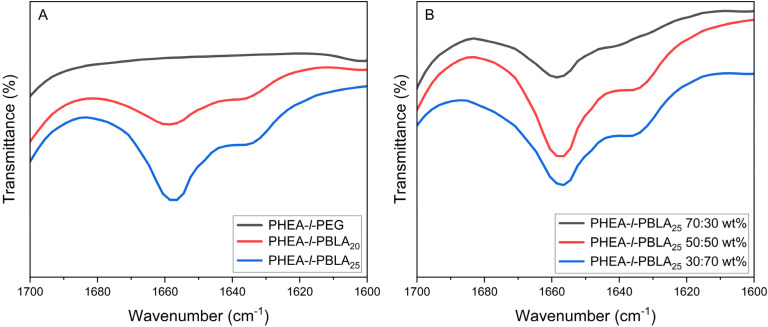
ATR-FTIR spectra of PHEA-*l*-(PBLA-*b*-PEG-*b*-PBLA) and PHEA-*l*-PEG conetworks in the amide I region from 1600–1700 cm^−1^. (A) PHEA-*l*-PBLA_20_-*b*-PEG-*b*-PBLA_20_, PHEA-*l*-PBLA_25_-*b*-PEG-*b*-PBLA_25_ and PHEA-*l*-PEG, with a 50 : 50 wt% ratio of peptide to PHEA. (B) Comparison of different compositions of PHEA-*l*-PBLA_25_-*b*-PEG-*b*-PBLA_25_ networks (30 : 70, 50 : 50 and 70 : 30 wt%).

The presence of α-helices and β-sheets in the material formed by the peptides was investigated using ATR-FTIR spectroscopy. The peaks between 1600 and 1700 cm^−1^ show the secondary structure of the peptides in the reinforced hydrogels, as previously reported in the literature.^24,35^ The amide bands in the ATR-FTIR spectrum between 1620 and 1645 cm^−1^ can be assigned to β-sheets, and the peak between 1650 and 1660 cm^−1^ can be attributed to α-helices.^24,35^Fig. 2A compares the peptidic and non-peptidic networks at a 50 : 50 wt% composition. The peaks corresponding to the α-helices are slightly more pronounced than those for the β-sheets, and the control PEG sample, which contains no peptides, does not show any peaks in this region, as expected. In Fig. 2B, which compares different compositions of networks with 30 : 70, 50 : 50, and 70 : 30 wt% ratios of peptide to comonomer, the spectra again exhibit characteristic peaks of α-helices and β-sheets. The peaks corresponding to α-helices are more predominant in all material compositions, regardless of the amount of peptide incorporated.

#### 2.2.2 Wide-angle X-ray scattering (WAXS)

**Fig. 3:**
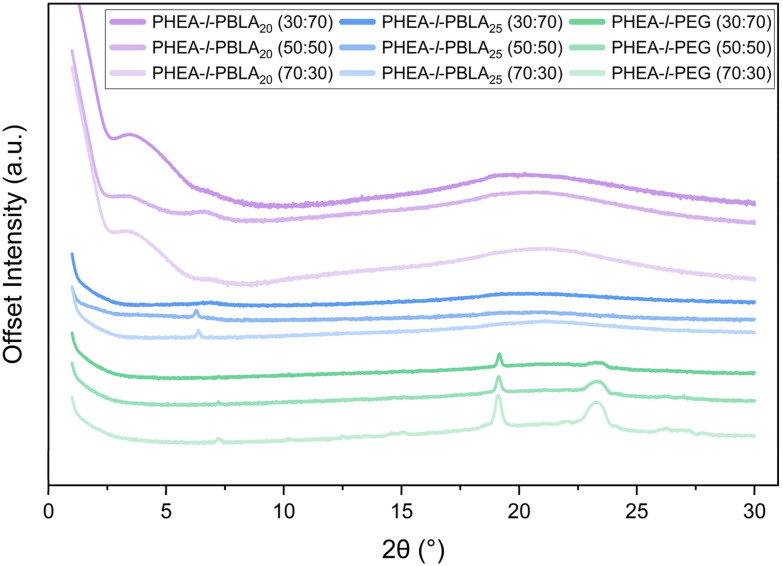
WAXS spectra of PHEA-*l*-(PBLA-*b*-PEG-*b*-PBLA) and PHEA-*l*-PEG conetworks.

WAXS measurements were carried out to further investigate the secondary structure formation within the peptide hybrid networks (Fig. 3). According to literature, characteristic features for the constituent materials were expected: an amorphous halo from 2*θ* = 15–25° for PHEA, crystalline peaks for PEG at 2*θ* = 19.2° and 23.2°, and for PBLA signals corresponding to β-sheets (∼5°) and α-helices (6°, 11°, and 12°).^25^ For the PHEA-*l*-PBLA_20_ sample, a mixture of peaks corresponding to both β-sheet and α-helix structures was observed, consistent with the ATR-FTIR results above and with previous findings that peptide segments at this peptide block length form a mixture of β sheets and α helices, with α-helical structures preferred.^25^ The PHEA-*l*-PBLA_25_ samples displayed predominantly α-helical signals with only very weak features in the β-sheet region, suggesting that the increased peptide length favours α-helix formation. Both peptide-containing samples exhibited a broad amorphous halo overlapping the region where the PEG peaks were expected to be observed, supporting the AFM and DSC data, which indicate suppressed PEG crystallisation and higher network homogeneity. In the PHEA-*l*-PEG control samples, no features were detected in the regions associated with peptidic secondary structures. However, a faint peak at ∼7° is observed, which may correspond to larger periodic structures or lamellar crystallinity of PEG, as seen during AFM imaging. As expected, clear PEG crystalline peaks at 19.2° and 23.2° were observed on top of the amorphous PHEA halo from 15–25°. Overall, the WAXS data provide clear evidence of peptide secondary structure formation in PBLA-containing networks, aligning with the ATR-FTIR data, and highlight the structural differences between peptidic and non-peptidic samples.

### 2.3 Morphological characterisation

#### 2.3.1 Atomic force microscopy (AFM)

**Fig. 4:**
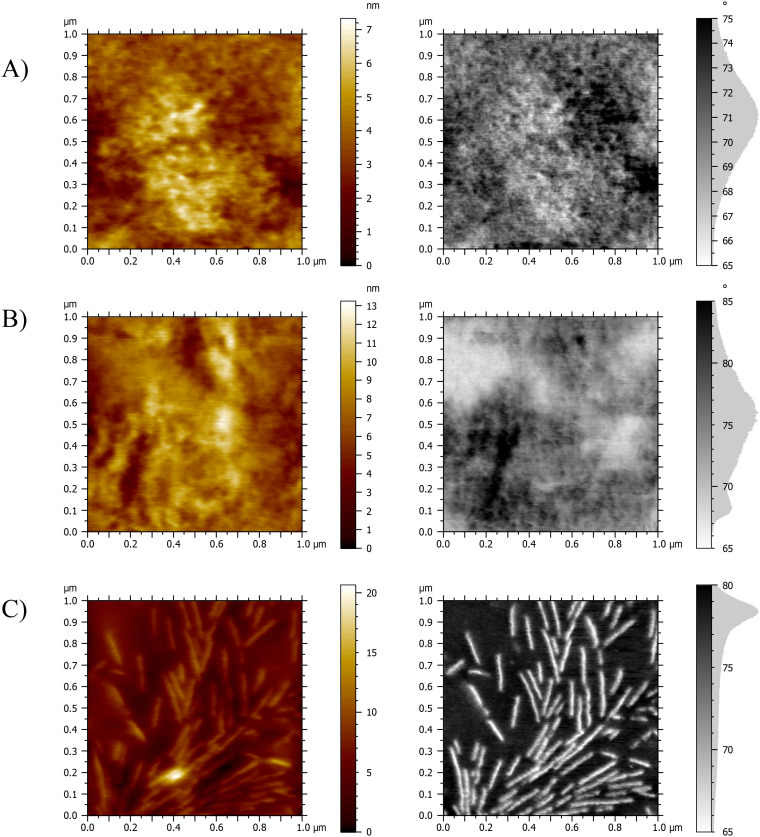
Characterisation of the morphology of dry PHEA-*l*-(PBLA-*b*-PEG-*b*-PBLA) and PHEA-*l*-PEG conetworks by AFM. Topographic and phase mode images of (A) PHEA-*l*-PBLA_20_ (50 : 50 wt%). (B) PHEA-*l*-PBLA_25_ (50 : 50 wt%). (C) PHEA-*l*-PEG (50 : 50 wt%).

AFM was employed to examine the morphology of the hydrogel networks and to gain insight into potential microscale phase separation within the material. The topographic and phase-mode AFM images of the cross-section of the films are displayed in Fig. 4. Fig. 4A and B show the 50 : 50 wt% compositions of the peptidic PHEA-*l*-(PBLA-*b*-PEG-*b*-PBLA) networks, and Fig. 4C shows the PHEA-*l*-PEG networks. The AFM images show relatively homogeneous samples with both hard and soft domains. The lighter areas represent low phase/hard material, indicating PEG phases with higher stiffness throughout the material, where crystallisation is inhibited by the peptide domains as reported by Jang and colleagues.^34^ The darker, high-phase areas represent softer regions, which are attributed to the PHEA and peptides.^34^ In the images of the PHEA-*l*-PEG, there is a much starker contrast between the phases, and it can be seen clearly throughout all compositions that crystallisation of the PEG has occurred, mostly in the form of spherulites, which is typical for PEG polymers.^36,37^ The remaining AFM images can be found in the SI (Fig. S5–10).

### 2.4 Thermal analysis

#### 2.4.1 Differential scanning calorimetry (DSC)

**Fig. 5:**
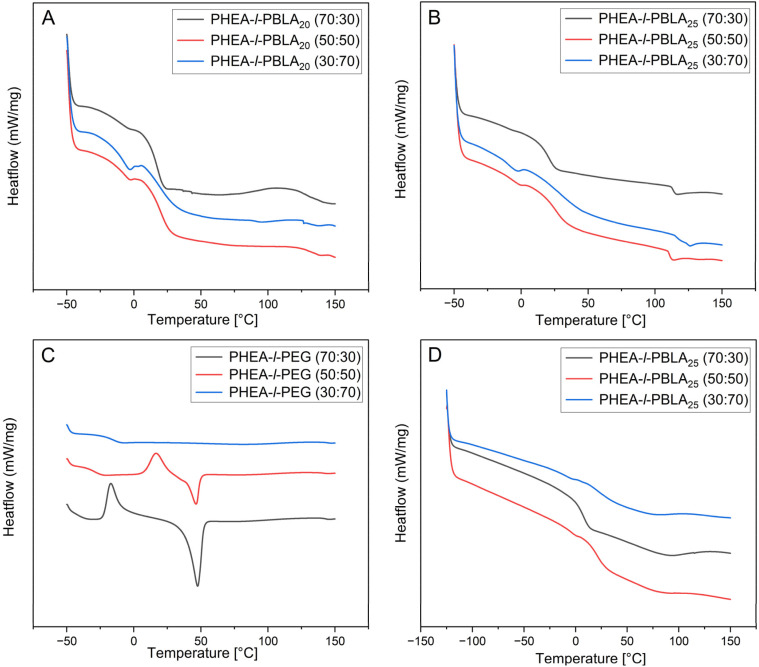
DSC thermograms (exothermic up) of PHEA-*l*-(PBLA-*b*-PEG-*b*-PBLA) and PHEA-*l*-PEG conetworks. (A) PHEA-*l*-PEG. (B) PHEA-*l*-PBLA_25_. (C) PHEA-*l*-PBLA_20_ measured from −50 °C to 150 °C. (D) PHEA-*l*-PBLA_25_ measured from −125 °C to 150 °C. All thermograms show the second heating cycle.

DSC analysis was conducted to investigate the thermal behaviour of the networks and to examine the influence of peptide incorporation on their thermal properties (Fig. 5, Table 1). As expected for multicomponent polymer systems, the materials exhibited multiple distinct thermal transitions corresponding to their constituent polymers. The literature values for the glass transition temperatures (*T*_g_) of PHEA, PBLA, and PEG are approximately −15 °C, 44 °C, and between −60 °C to 20 °C, respectively, with PEG's *T*_g_ varying widely depending on molecular weight and crystallinity.^38–40^ These values are strongly influenced by polymer composition and intermolecular interactions, especially within crosslinked networks. In peptide-containing networks, the glass transitions of the individual components shifted to higher temperatures, and the transitions of PHEA and PBLA overlap, likely due to the miscibility between the peptide and PHEA phases, which was reported previously in PHEA-*l*-PDMS networks reinforced with PBLA (Fig. 5A and B).^25^ The glass transitions attributed to the PEG domains were between −5 and −12 °C in the peptide-containing networks. In contrast, the non-peptidic PEG networks exhibited significantly lower *T*_g_ values, ranging from −27 to −15 °C (Fig. 5C). The upward shift of *T*_g_ suggests reduced segmental mobility of the PEG chains in the presence of PBLA blocks. This restriction is likely due to additional physical crosslinks introduced by peptide–peptide interactions and secondary structure formation, which constrain the local motion of PEG segments and thereby raise the *T*_g_. Similar effects of α-helical and β-sheet ordering on thermal transitions have been reported in peptide–polymer hybrids, where hydrogen bonding and peptidic ordering suppress PEG crystallisation and shift thermal transitions to higher temperatures.^34^ The *T*_g_ associated with PHEA ranged from 6 °C to 27 °C, with a trend of increasing *T*_g_ as the PHEA content decreased. Additionally, the *T*_g_s in this region occur around room temperature and will likely affect the mechanical properties of the networks. Broad thermal transitions around 110 °C were observed in the peptidic networks, potentially corresponding to the disruption of α-helical and β-sheet structures formed by the peptides, or to other non-covalent interactions within the network. To better understand the behaviour of PEG in the network, DSC analysis of the PHEA-*l*-PBLA_25_-*b*-PEG-*b*-PBLA_25_ network was extended down to −125 °C (Fig. 5D). However, no glass transition temperatures were observed in this extended lower range, likely due to disrupted PEG crystallinity and restricted chain mobility resulting from crosslinking and peptide-induced interactions. Overall, the peptidic networks exhibit multiple glass transitions corresponding to their different components, consistent with a multiphasic network. The suppression of crystallisation and melting transitions in these samples supports the hypothesis that peptide secondary structures disrupt PEG crystallisation, as also suggested by AFM imaging.

**Table 1:** Summary of thermal transitions observed in DSC thermograms of PHEA-*l*-(PBLA-*b*-PEG-*b*-PBLA) and PHEA-*l*-PEG conetworks

Sample	*T*_g_ (°C)			*T*_*exo*_ (°C)	*T*_*endo*_ (°C)
	PEG	PHEA	PBLA		
PHEA-*l*-PEG (70 : 30)^a^	—	—	—	−17	47
PHEA-*l*-PEG (50 : 50)^a^	−27	—	—	8	46
PHEA-*l*-PEG (30 : 70)^a^	−15	—	—	—	—
PHEA-*l*-PBLA_20_ (70 : 30)^a^	−12	6	—	—	130
PHEA-*l*-PBLA_20_ (50 : 50)^a^	−9	19	—	—	135
PHEA-*l*-PBLA_20_ (30 : 70)^a^	−10	18	—	—	130
PHEA-*l*-PBLA_25_ (70 : 30)^a^	−5	20	—	—	112
PHEA-*l*-PBLA_25_ (50 : 50)^a^	−7	25	—	—	110
PHEA-*l*-PBLA_25_ (30 : 70)^a^	−9	27	—	—	117
PHEA-*l*-PBLA_25_ (70 : 30)^b^	6	—	—	—	100
PHEA-*l*-PBLA_25_ (50 : 50)^b^	−8	21	—	—	100
PHEA-*l*-PBLA_25_ (30 : 70)^b^	−8	33	—	—	100

a DSC measurements from −50 °C to 150 °C.

b DSC measurements from −125 °C to 150 °C.

In the non-peptidic PHEA-*l*-PEG networks with 30 and 50 wt% PEG, clear crystallisation and endothermic events (*T*_*exo*_ = −17 and 8 °C and *T*_*endo*_ = 47 and 46 °C, respectively) were registered. These endothermic melting events align with the melting of the crystalline PEG domains that were observed by AFM. These transitions were absent in the 70 wt% PEG network. The measurement was repeated in triplicate, and no events were observed in the first or second heating cycle. Suppressed events are possibly due to lower bulk crystallinity compared to other compositions, and due to the higher crosslinking density in the 70% PEG composition. Perhaps the transition is very small in enthalphy and spread over a broad temperature range, or partially overlaps and cancels in the heat-flow signal. This response could also be because the heating/cooling rate of the DSC measurement occurs on a much shorter timescale than the crystallisation process of the PEG. Glass transitions of PEG were also observed in the PHEA-*l*-PEG control networks at −15 °C and −27 °C for the 50 and 70 wt% samples, respectively. In the PHEA-*l*-PEG 70 : 30 wt% network, no clear *T*_g_ was observed, which is likely due to overlap with the exothermic event at −17 °C.

#### 2.4.2 Thermal gravimetric analysis (TGA)

**Fig. 6:**
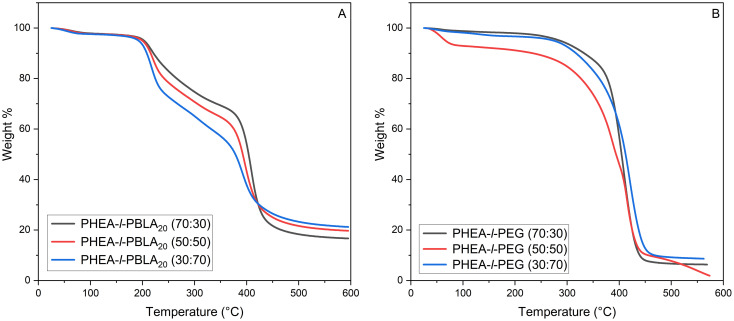
TGA thermograms of (A) PHEA-*l*-(PBLA-*b*-PEG-*b*-PBLA) and (B) PHEA-*l*-PEG conetworks.

TGA analysis was performed to assess the thermal stability and degradation of the conetworks (Fig. 6). For the peptide-containing samples, a clear two-stage degradation is observed, with the initial loss occurring at ∼200 °C and a second sharp drop-off at 400 °C. This initial mass loss at 200 °C is likely due to thermal decomposition of PBLA, which increases with increasing peptide content. This transition is followed by the degradation of the remaining material at higher temperatures. The non-peptidic PHEA-*l*-PEG samples exhibit a single degradation event, with an onset temperature around 250 °C and a sharp drop in mass at around 400 °C. This behaviour indicates that the non-peptidic material is thermally stable until around 250 °C, which is comparable to the degradation of a PEG homopolymer with a molecular weight of 4000 g mol^−1^ at 255 °C reported by Li *et al.*^39^ In all samples, there is a slight initial weight drop, likely due to residual moisture in the materials. In the PHEA-*l*-PEG (50 : 50 wt%) sample, there is a more significant initial loss in weight. This behaviour could arise from trapped residual DMSO and bound water that remains in the hydrophilic PEG/PHEA network after drying. There may also possibly be minor contributions from trapped low-molecular-weight polymer and photoinitiator fragments.

### 2.5 Swelling behaviour

**Fig. 7:**
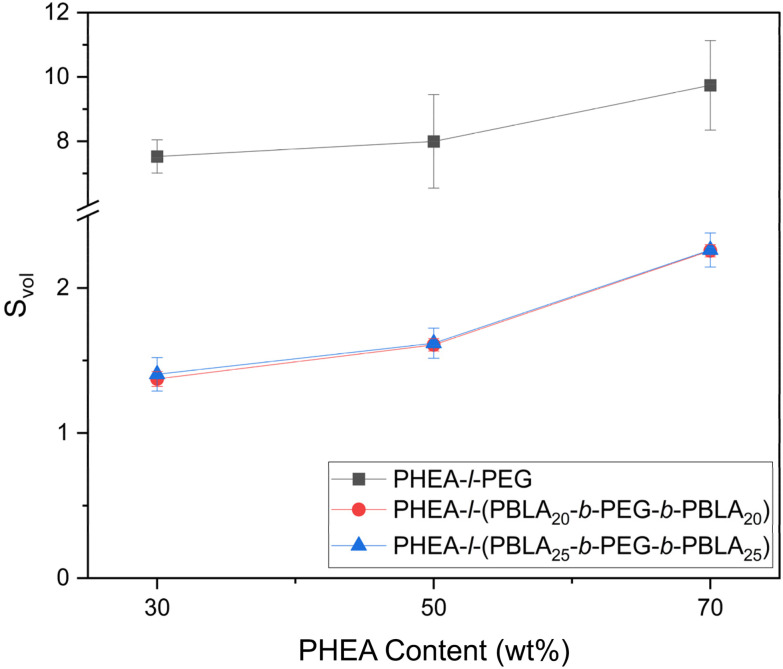
Comparison of the swelling in water of the peptidic PHEA-*l*-(PBLA-*b*-PEG-*b*-PBLA) conetworks as a function of PHEA content to the swelling of the non-peptidic PHEA-*l*-PEG conetworks.

One of the key properties of lightly crosslinked polymer networks is their ability to swell in solvents, *i.e.*, water in the case of hydrogels. We therefore investigated the effect of PBLA incorporation and block length on the aqueous swelling behaviour of the networks presented herein. Across both peptide-containing and non-peptidic hydrogels, an increase in PHEA content resulted in higher swelling ratios, with the materials containing 70 wt% PHEA displaying the greatest swelling (Fig. 7, Table 2). This swelling response is consistent with higher hydrophilicity and lower crosslinking density within these networks. As the peptide blocks are slightly hydrophobic and add additional physical crosslinking points to the network, the peptide-containing networks swelled less than the PHEA-*l*-PEG networks. However, the peptide-containing materials nonetheless exhibited marked swelling in water, from a swelling ratio *S*_vol_ = 1.37 ± 0.05 at 30 wt% PHEA content to *S*_vol_ = 2.26 + 0.04 at 70% PHEA content, independent of the length of the peptide segments. Thus, despite the presence of more hydrophobic peptide segments, water was still able to diffuse into the network through the PEG and PHEA domains.

**Table 2:** Summary of swelling data of PHEA-*l*-(PBLA-*b*-PEG-*b*-PBLA) and PHEA-*l*-PEG conetworks. Average of *n* = 3 measurements ± standard deviation (SD)

Sample	Swelling ratio (*S*_vol_)
PHEA-*l*-PEG (70 : 30)	7.53 ± 0.52
PHEA-*l*-PEG (50 : 50)	7.99 ± 1.46
PHEA-*l*-PEG (30 : 70)	9.74 ± 1.39
PHEA-*l*-PBLA_20_ (70 : 30)	1.37 ± 0.05
PHEA-*l*-PBLA_20_ (50 : 50)	1.61 ± 0.04
PHEA-*l*-PBLA_20_ (30 : 70)	2.26 ± 0.04
PHEA-*l*-PBLA_25_ (70 : 30)	1.40 ± 0.11
PHEA-*l*-PBLA_25_ (50 : 50)	1.62 ± 0.10
PHEA-*l*-PBLA_25_ (30 : 70)	2.26 ± 0.12

### 2.6 Mechanical properties of conetworks

#### 2.6.1 Tensile testing

**Fig. 8:**
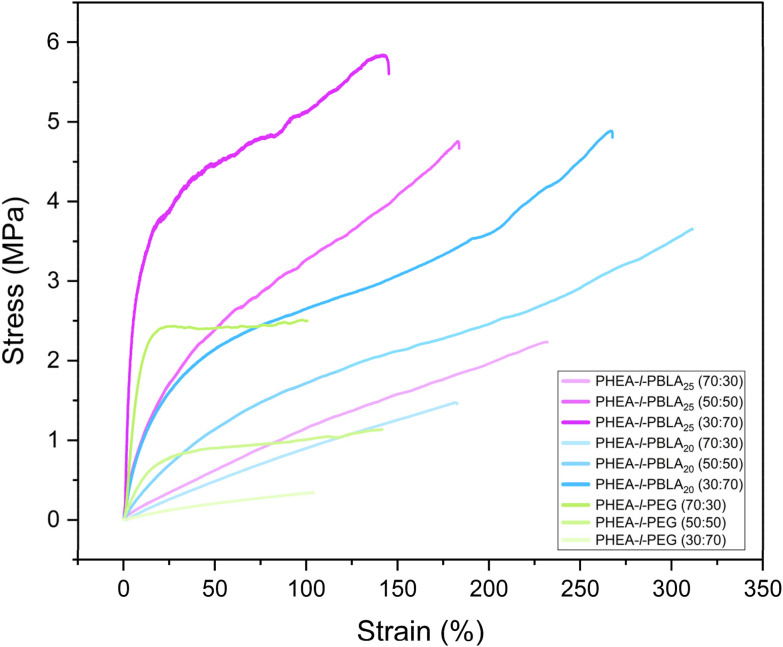
Representative stress–strain curves from uniaxial tensile tests of dry PHEA-*l*-(PBLA-*b*-PEG-*b*-PBLA) conetworks and dry PHEA-*l*-PEG conetworks.

Tensile testing was performed to assess the mechanical properties of the dry polymer networks, revealing a clear influence of peptide incorporation (Fig. 8, Table 3). The extensibility of the networks, as measured by the maximum strain, was significantly enhanced by the peptide blocks. For example, the PHEA-*l*-PBLA_20_ 50 : 50 wt% sample reached 308 ± 40%, whereas the PHEA-*l*-PEG 50 : 50 wt% samples exhibited a maximum strain of 139 ± 41%. In terms of mechanical strength, the maximum stress of the peptide-containing networks increased with the peptide content: from 1.97 ± 0.28 MPa for 70 : 30 wt% to 5.43 ± 0.44 MPa for 30 : 70 wt% for PHEA-*l*-PBLA_25_ samples. In contrast, the maximum stress of the non-peptidic PHEA-*l*-PEG networks decreased with increasing PEG crosslinker content, lowering from 2.77 ± 0.38 MPa at 70 : 30 wt% to 0.35 ± 0.09 MPa at 30 : 70 wt%.

**Table 3:** Summary of tensile experimental data of PHEA-*l*-(PBLA-*b*-PEG-*b*-PBLA) and PHEA-*l*-PEG conetworks. Average of *n* = 8 measurements ± SD

Sample	Young's modulus (MPa)	Toughness (MJ m^−3^)	Max. stress (MPa)	Max. strain (%)
PHEA-*l*-PEG (70 : 30)	31.7 ± 4.5	2.8 ± 2.2	2.8 ± 0.4	113 ± 73
PHEA-*l*-PEG (50 : 50)	4.6 ± 1.2	1.3 ± 0.5	1.2 ± 0.3	139 ± 41
PHEA-*l*-PEG (30 : 70)	0.55 ± 0.04	0.2 ± 0.1	0.35 ± 0.1	109 ± 33
PHEA-*l*-PBLA_20_ (70 : 30)	1.26 ± 0.14	0.6 ± 0.1	0.7 ± 0.3	181 ± 37
PHEA-*l*-PBLA_20_ (50 : 50)	3.4 ± 0.6	5.5 ± 1.2	3.4 ± 1.5	308 ± 39
PHEA-*l*-PBLA_20_ (30 : 70)	11.0 ± 1.6	7.7 ± 2.6	4.9 ± 1.4	261 ± 43
PHEA-*l*-PBLA_25_ (70 : 30)	4.4 ± 1.9	2.4 ± 0.3	2.0 ± 0.3	224 ± 13
PHEA-*l*-PBLA_25_ (50 : 50)	29.6 ± 8.4	4.4 ± 0.8	4.2 ± 0.8	165 ± 14
PHEA-*l*-PBLA_25_ (30 : 70)	125 ± 15	6.9 ± 0.7	5.4 ± 0.4	145 ± 11

A similar trend was observed for the Young's modulus, which increased significantly with peptide incorporation. The PHEA-*l*-PBLA_25_ 70 : 30 wt% exhibited a Young's modulus of 4.45 ± 1.94 MPa, and the one of PHEA-*l*-PBLA_25_ 30 : 70 wt% was 125 ± 15 MPa, increasing by a factor of 28. In contrast, in the non-peptidic samples, the Young's modulus decreased with increasing PEG content from 31.79 ± 4.52 MPa in the PHEA-*l*-PEG 70 : 30 wt% to only 0.55 ± 0.04 MPa in PHEA-*l*-PEG 30 : 70 wt%. Toughness followed a similar pattern: non-peptidic networks became less tough as the PEG crosslinker content increased, while peptide-containing networks became tougher with a greater peptide content. These results collectively suggest that the peptides reinforce the network and enhance their ability to dissipate energy under stress, thereby improving both strength and extensibility and, as a result, toughness.

There is a noticeable and significant difference in the mechanical properties between peptidic networks with 20 and 25 PBLA repeat units. The Young's modulus in the 50 : 50 wt% networks exhibited an increase from 3.41 ± 0.58 MPa in PHEA-*l*-PBLA_20_ to 29.6 ± 8.41 MPa in PHEA-*l*-PBLA_25_. The difference between the samples is 5 repeat units on either side of the central PEG block, resulting in 10 additional repeat units of benzyl-l-aspartate per crosslinker. The longer segments in the 25-repeat unit system increase the chain length between crosslinking points, allowing for greater chain flexibility, entanglement, and a higher potential for intermolecular interactions. These include the formation of ordered secondary structures, hydrophobic clustering, and physical associations that enhance network cohesion. Thus, the additional repeat units increase the crosslinker chain length, enabling more extensive non-covalent crosslinking and reinforcing the mechanical integrity of the network through improved stress dissipation and network connectivity.

#### 2.6.2 Rheology of dry conetworks and hydrogels

**Fig. 9:**
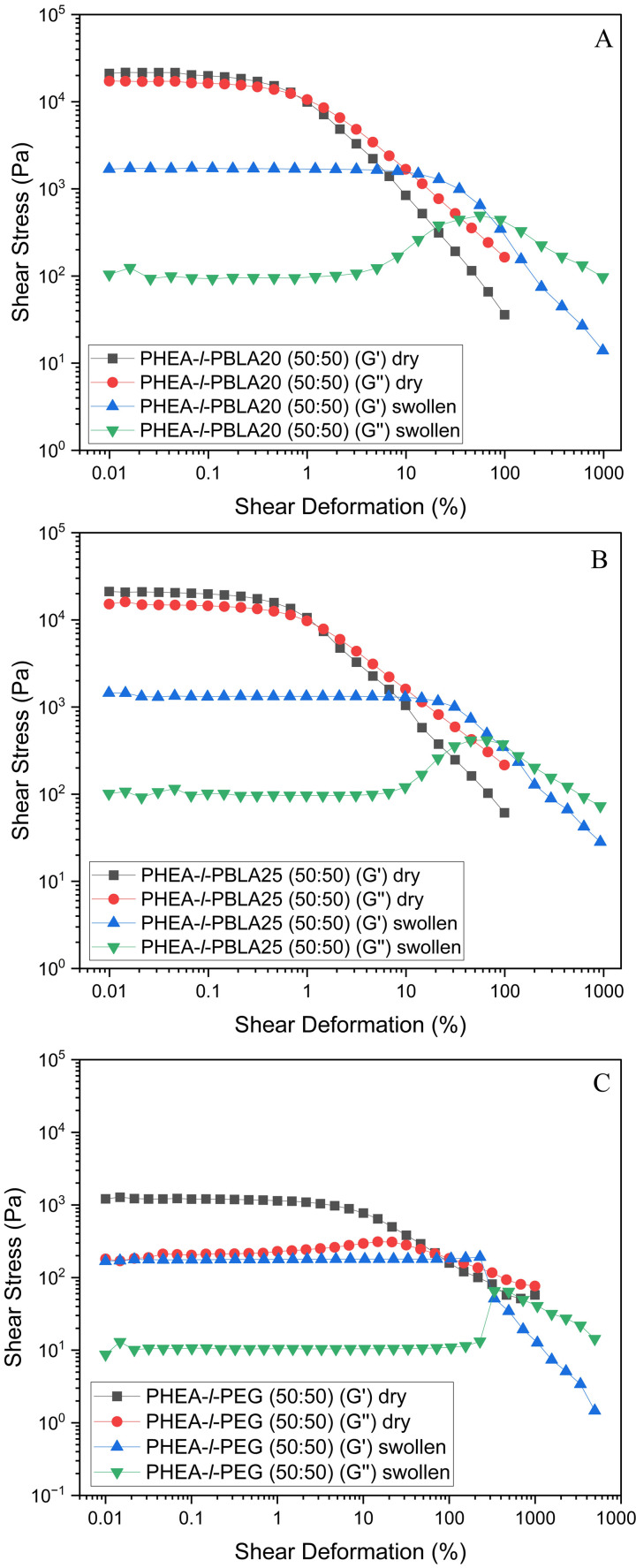
Rheology data of dry and water-swollen PHEA-*l*-(PBLA-*b*-PEG-*b*-PBLA) and PHEA-*l*-PEG conetworks with a 50 : 50 wt% composition. (A) PHEA-*l*-PBLA_20_, (B) PHEA-*l*-PBLA_25_, and (C) PHEA-*l*-PEG.

Rheological measurements were performed on the networks to characterise their viscoelastic properties both in the dry state and when swollen in water. Fig. 9 shows the rheology results for the amplitude sweep of the networks with 50 : 50 wt% composition, where *G*′ is the storage modulus and *G*″ is the loss modulus. The rheology measurements for the remaining samples are provided in the SI (Fig. S9). In the dry state, the peptidic networks (PHEA-*l*-PBLA_20_ and PHEA-*l*-PBLA_25_) generally displayed improved mechanical properties when compared to the non-peptidic networks. For example, the PHEA-*l*-PBLA_20_ network at 50 : 50 wt% exhibited the highest dry-state storage modulus (*G*′ = 21 570 Pa), with PHEA-*l*-PBLA_25_ (50 : 50 wt%) following closely at *G*′ = 19 610 Pa. These values far exceed those of the non-peptidic PHEA-*l*-PEG network with 50 : 50 wt% composition (*G*′ = 1200 Pa in the dry state), indicating that the presence of peptides enhances the network stiffness in the dry state through additional physical crosslinks. Notably, in the dry state, the peptidic networks with compositions of 30 : 70 and 50 : 50 wt% show that *G*′ > *G*″ in the linear viscoelastic region (Fig. 9 and Fig. S9), *i.e.*, they display gel-like character. After exceeding this linear-viscoelastic (LVE) range, the network structures collapsed. The occurrence of an intersection point *G*′ = *G*″ indicates macrocracks forming in the cross-linked polymer.^41^ In contrast, for the 70 : 30 wt% peptidic samples, *G*″ is larger than *G*′ over the entire shear deformation range tested, and the storage and loss moduli do not intersect, which suggests that the material behaves predominantly as a viscous fluid rather than a solid.

According to the DSC data (Table 1), the *T*_g_ of PHEA is close to the measurement temperature (25 °C). Near *T*_g_, *G*′ decreases significantly while *G*″ increases and often exhibits a maximum, indicating efficient energy dissipation. Under these conditions, damping is maximised, and loss-dominated behaviour (*G*″ > *G*′) within the LVE region is favoured. In contrast, the *T*_g_ of the PEG block lies well below the measurement temperature, imparting a pronounced viscous contribution. Due to crosslinking, this viscous component is embedded within an overall elastic network. Maximum damping, associated with *G*″>*G*′, occurs when the fraction of the dissipative PHEA block, whose *T*_g_ is close to the measurement temperature, is sufficient to counterbalance the elastic restoring capacity of the PEG block while maintaining substantial segmental mobility. This condition is met in the PHEA-*l*-PBLA_20_ (70 : 30) and PHEA-*l*-PBLA_25_ (70 : 30) samples. When the dissipative block is present in too small a fraction, storage-dominated behaviour prevails; in contrast, when it is present in excess, the material loses its solid-like character and approaches liquid-like behaviour.

Upon swelling in water, the *G*′ and *G*″ of the networks dropped, but the PBLA-containing hydrogels maintain substantial mechanical integrity (Fig. 9, Fig. S9). For instance, PHEA-*l*-PBLA_20_ and PHEA-*l*-PBLA_25_ (50 : 50 wt%) retained a swollen-state *G*′ of 1670 Pa and 1311 Pa, respectively, compared to only 141 Pa for their non-peptidic PHEA-*l*-PEG counterpart. Interestingly, samples with an intermediate PBLA content of 50 wt% displayed the highest *G*′ values, suggesting that this composition provides the most robust network, with optimal stiffness and firmness. These results demonstrate that the peptide segments reinforce the peptide-containing networks also in hydrated conditions. In the swollen state, all samples showed a distinct peak in the loss modulus, *G*″, indicating that the materials absorbed and dissipated energy before they fully yielded. Interestingly, there are also peaks in the *G*″ of the dry PHEA-*l*-PEG networks, suggesting energy dissipation in the network due to the crystalline PEG domains.

The loss tangent tan *δ* = *G*″/*G*′ of the networks in the LVE region decreased significantly by swelling the polymers (Fig. 9, Table 4). For example, the tan *δ* of the PHEA-*l*-PBLA_20_ network (50 : 50 wt%) lowered from 0.79 Pa in the dry state to 0.06 Pa when swollen. When comparing the peptidic networks, tan *δ* decreased with increasing PHEA content and was lower for PHEA-*l*-PBLA_25_ than for PHEA-*l*-PBLA_20_. The lower tan *δ* values indicate a more elastic behaviour, possibly indicating reduced molecular mobility and increased structural order. Together, these results clearly demonstrate improved mechanical performance of the hydrogel networks when peptidic segments are incorporated. The data indicate that interactions between the PBLA peptides, such as hydrogen bonding and secondary structure formation, help maintain the network's cohesion under hydrated conditions and dissipate energy upon deformation. Thus, PBLA-reinforced PEG-based networks represent viable alternatives to traditional PEG networks in terms of robust mechanical performance, particularly when swollen in water, *i.e.* when used as hydrogels.

**Table 4:** Summary of rheology data of PHEA-*l*-(PBLA-*b*-PEG-*b*-PBLA) and PHEA-*l*-PEG conetworks

Sample	Storage modulus *G*′ (Pa)		Loss modulus *G*″ (Pa)		Loss factor tan *δ* (*G*′/*G*″)	
	Dry	Swollen	Dry	Swollen	Dry	Swollen
PHEA-*l*-PEG (70 : 30)	4520	141	650	10.4	0.14	0.074
PHEA-*l*-PEG (50 : 50)	1200	177	204	10.4	0.17	0.060
PHEA-*l*-PEG (30 : 70)	800	300	210	73.1	0.26	0.024
PHEA-*l*-PBLA_20_ (70 : 30)	3240	1590	3730	46.7	1.15	0.029
PHEA-*l*-PBLA_20_ (50 : 50)	21 570	1670	17 190	99.7	0.79	0.060
PHEA-*l*-PBLA_20_ (30 : 70)	16 130	630	7510	91.8	0.47	0.146
PHEA-*l*-PBLA_25_ (70 : 30)	3490	550	3720	22.9	1.1	0.042
PHEA-*l*-PBLA_25_ (50 : 50)	19 610	1310	14 330	99.5	0.73	0.076
PHEA-*l*-PBLA_25_ (30 : 70)	1530	1140	430	143	0.28	0.126

## 3. Conclusion

Macromolecular crosslinkers with a central PEG block, adjacent PBLA blocks, and methacrylate end groups were synthesised and used to crosslink HEA by UV-induced, free radical polymerisation, resulting in PHEA-*l*-(PBLA-*b*-PEG-*b*-PBLA) conetworks in which the PHEA-to-crosslinker composition could be varied over a broad composition range by adjusting the ratio of monomer to macro-crosslinker in the reaction mixture. Swelling of the networks was achieved in water, and the resulting hydrogels showed enhanced mechanical properties compared to their non-peptidic counterparts. The PBLA segments between the crosslinking points and the PEG segments reinforced the materials in both the dry and hydrogel states by introducing physical crosslinks in addition to the chemical crosslinks inherent to the network architecture. Moreover, the mechanical properties of the materials could be tuned by varying the length of the incorporated peptide and adjusting the monomer-to-crosslinker ratio. Overall, this work presents a bio-inspired approach to polymer network and hydrogel reinforcement, demonstrating that incorporating peptides into PEG-based hydrogels can enhance mechanical properties while preserving the classic properties of hydrogels, including their ability to swell in water. This new class of hydrogel materials could find applications as biomedical hydrogels, such as tissue engineering scaffolds or drug delivery vehicles. Moreover, the PBLA-*b*-PEG-*b*-PBLA crosslinkers with methacrylate groups installed on both chain ends also have potential as photocurable bioinks for 3D printing with built-in mechanical tunability.

## 4. Materials

Commercial α,ω-methacrylate-terminated poly(ethylene glycol) (MA-PEG-MA; MW = 4000 g mol^−1^) was purchased from Creative PEGworks. Isocyanoethyl methacrylate, dibutyltin dilaurate, 2-hydroxyethyl acrylate, triethylamine, 2-hydroxy-4′-(2-hydroxyethoxy)-2-methylpropiophenone (Irgacure 2959), and *N*,*N*-dimethylacetamide (DMAc, anhydrous, 99.8%, packaged under argon), tetrahydrofuran (THF, anhydrous, >99.9%), β-benzyl-l-aspartate (BLA), and triphosgene, were purchased from Sigma-Aldrich. All solvents and reagents listed above were used as received. α,ω-Bis(amine)poly(ethylene glycol) (PEG, 2000 g mol^−1^) was purchased from Sinopeg (China) and was dried under vacuum at ∼80 °C for 3 h and then at room temperature for 16 h prior to use. β-Benzyl-l-aspartate *N*-carboxyanhydride (BLA–NCA) was synthesised according to the established literature procedure.^42^ Adhesive polypropylene tape (50 µm thickness) was obtained from Tesa, Germany.

## 5. Methods

### 5.1 Synthesis of PBLA-*b*-PEG-*b*-PBLA

PBLA-*b*-PEG-*b*-PBLA triblock copolymers were synthesised *via* amine-initiated NCA ring-opening polymerisation in a nitrogen (N_2_) atmosphere glovebox, as previously reported.^34^ The ratio of NCA monomer to PEG diamine was controlled to target specific peptide repeat lengths (*e.g.* 25 peptide units per block). As an example, BLA–NCA (25 mmol), and a mixture of THF and DMAc in a volumetric ratio of 1 : 3 (31 mL) was added to an oven-dried, 100 mL round-bottom flask equipped with a magnetic stirrer and a Vigreux condenser. To the BLA–NCA solution, 0.5 mmol of PEG predissolved in 29 mL of 1 : 4 THF : DMAc solution was added. The mixture was stirred at 22 °C for 24 hours. The product polymer was precipitated into diethyl ether, collected *via* vacuum filtration, washed with diethyl ether three times, and dried under vacuum at 22 °C overnight.

### 5.2 Chain end modification with methacrylate groups

The chain ends of PBLA-*b*-PEG-*b*-PBLA were modified with methacrylate (MA) groups by reaction with isocyanoethyl methacrylate. The triblock copolymer (2.0 g) was mixed with THF (45 mL) as the solvent and 0.4 mL of isocyanato ethyl methacrylate (439 mg, 2.83 mmol). Dibutyltin dilaurate (552 µl, 580 mg, 0.92 mmol) was added dropwise. The reaction was stirred at room temperature overnight. The solvent was removed using a rotary evaporator. The resulting product was placed in a vacuum oven to dry overnight at 50 °C and 100 mbar.

### 5.3 Synthesis of polymer networks

Polymer networks of varying compositions of HEA and the crosslinkers were synthesised by UV-initiated free radical polymerisation. Accordingly, polyethylene glycol dimethacrylate (MA-PEG-MA) or the peptide-containing crosslinkers MA-PBLA-*b*-PEG-*b*-PBLA-MA were dissolved in DMSO at room temperature using a vortexer. Once dissolved, HEA was added, and the mixture was vortexed further. The photo-initiator was then added to the mixture in powder form, and the mixture was vortexed again to dissolve the initiator. In a typical sample preparation (*e.g.* 50 : 50 wt% PHEA-*l*-(PBLA-*b*-PEG-*b*-PBLA)), 200 mg of MA-PBLA-*b*-PEG-*b*-PBLA-MA was dissolved in 600 µL of DMSO and vortexed until dissolved, 200 µL of HEA was then added and further vortexed before 8 mg of Irgacure 2959 was added. Approximately 400 µL of the solution was then added to a microscope slide, which was covered with one layer of Tesa tape across the entire slide, and had stacked layers of Tesa tape as spacers at the edges, resulting in a final thickness of ∼200 µm. Another glass slide covered in the same tape was placed on top, and the whole setup was placed inside a UV curing lamp (Dymax 500) and irradiated for 18 min, turning the sample after 9 min had elapsed. The resulting polymer network film sample was carefully removed from the glass slide and dried in a vacuum oven at 40 °C for at least 2 hours.

### 5.4 Swelling of hydrogels

The swelling behaviour of the networks was measured by immersing dry samples (approx. 6 mm × 10 mm in size) in distilled water overnight at room temperature. The edge length (*L*_i_) was measured for the dry and swollen samples using a Zeiss Stemi 508 optical microscope fitted with Axiocam 208 colour using reflected light mode. The swelling ratio, *S*_vol_, was determined from the edges of the sample as:
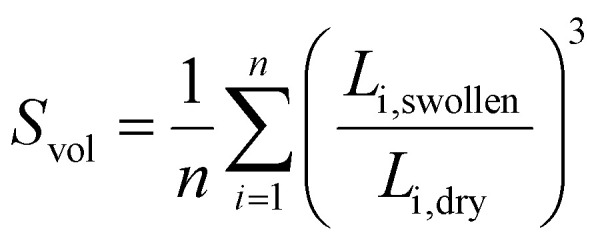
where *n* denotes the number of edges.

## 6. Instrumentation

### 6.1 ^1^H Nuclear magnetic resonance spectroscopy

For the NMR spectroscopy, approximately 10 mg of sample was dissolved in 750 µL CDCl_3_. ^1^H NMR spectroscopy was performed at room temperature on a Bruker Avance II spectrometer (Rheinstetten, Germany) operating at 300 MHz. NMR data was processed using MestReNova x64.

### 6.2 Gel permeation chromatography

For GPC measurements, the synthesised triblock copolymers were dissolved in the GPC solvent (THF) and shaken for several hours before filtration with a 0.45 µm poly(tetrafluoroethylene) (PTFE) filter. The measurements were carried out on Agilent 1260 Infinity instruments equipped with refractive index (RI) and UV-vis detectors. SDV linear-M columns were used for THF at 25 °C. Samples were run at a flow rate of 1 mL min^−1^. PSS ReadyCal-kit PS was used for calibrating the instrument. Number-average molecular weight (*M*_n_) and dispersity (*Đ*) values were determined using the PSS WinGPC software.

### 6.3 Attenuated total reflectance-Fourier transform infrared spectroscopy

ATR-FTIR spectroscopy was used to analyse the presence and content of β-sheets and α-helices in the peptide-reinforced hydrogels and the peptidic triblock copolymers using a Bruker α II ATR-FTIR spectrometer. All polymer network films were measured in absorbance mode with 128 scans and data was processed using OPUS software.

### 6.4 Wide angle X-ray scattering

WAXS measurements were performed using a Rigaku SmartLab diffractometer equipped with a Cu Kα radiation source (*λ* = 0.154 nm), operated in reflection mode. Scans were recorded over a 2*θ* range of 1° to 30°, with a step size of 0.01° and a scan rate of 1° min^−1^, under ambient conditions. Samples were measured as dry polymer films mounted on a flat sample holder. Data collection and processing were carried out using Rigaku SmartLab Studio II software.

### 6.5 Atomic force microscopy

Topography images and phase shift images were acquired in the air in tapping mode at scan rates ranging from 1 to 3 Hz with an image resolution of 512 × 512 pixels^2^ on an MFP3D INFINITY microscope (Oxford Instrument, UK). Semi-contact silicon AC160TS AFM tips (Olympus, Japan) with a cantilever spring constant of 26 N m^−1^ were used. Surface topography was acquired by maintaining the cantilever's first resonance amplitude constant *via* the feedback loop of the AFM acting on the piezo *Z* direction. The phase shift signal was recorded at the same time. Before the analysis, the hydrogel films were cross-sectioned and surfaced between −10 °C to 0 °C, using a UC6 Cryo-Ultramicrotome (Leica, Germany) equipped with a diamond blade (Diaotome, Switzerland). This preparation aims to obtain a representative image of the film's internal composition and to avoid possible skin effects. Data treatment was done with MountainSpip software (Digital Surf, France).

### 6.6 Thermogravimetric analysis

TGA measurements were conducted on a Mettler Toledo TGA 2 StarE system, and data were processed with Star E software. For sample preparation, approximately 10 mg of material was added to a pre-weighed open aluminium pan. The sample was then heated at a rate of 10 °C min^−1^ from 25 °C to 600 °C, during which time the change in sample weight was recorded. Nitrogen was used as the sample purge gas.

### 6.7 Differential scanning calorimetry

DSC measurements were conducted on a Mettler Toledo DSC 1 StarE system, and data were processed with Star E software. For sample preparation, approximately 5 mg of polymer film was weighed into an aluminium DSC pan and sealed non-hermetically with an aluminium lid. The sample pan was then loaded into an autosampler. The sample and reference were heated to 150 °C at a scan rate of 10 °C min^−1^, and the resulting heat flow response was monitored. The sample was re-cooled to −50 °C (or −125 °C) and then reheated again to 150 °C, all at a rate of 10 °C min^−1^. Nitrogen was used as the purge gas. The glass transition temperatures were determined from the transition mid-points of the second heating curve.

### 6.8 Tensile measurements

The mechanical properties of the hydrogels were measured using a Zwick 1.0 tensile machine with a 10 N load cell. In each case, a total of eight samples of each composition of conetworks were tested at a speed of 10 mm min^−1^. The samples were cut in a dog-bone shape following DIN 53504 S3 specifications with a sample thickness of approximately 0.2 mm. Measurements were taken in a temperature and humidity-controlled room, and all samples were left to equilibrate for at least 24 hours before measurements were taken. All tests were performed with dry samples at temperatures of around 25 °C and 50% relative humidity.

### 6.9 Rheology measurements

Rheology measurements were taken on an Anton Paar MCR 301 Rheometer fitted with a TrueGap measuring chamber with Peltier temperature control bonnet H-PTD-200 and a plate-plate 25 mm (PP25) measuring system using RheoCompass Version 1.31 software. An amplitude sweep of the samples was measured at 25 °C in oscillating mode with an angular frequency (omega) of 10 rad s^−1^ and a shear strain (gamma) range from 0.01% to 100, 1000, or 5000% depending on the sample. Samples were cut into discs of 5 mm diameter for analysis and allowed to equilibrate in ambient conditions for 24 h prior to measurement.

## Conflicts of interest

There are no conflicts to declare.

## Supplementary Material

LP-004-D5LP00335K-s001

## Data Availability

The datasets generated and analysed during the current study are openly available from the University of Strathclyde's Pure repository at https://doi.org/10.15129/23f17944-2004-4f74-9d47-b285091a57d5. Supplementary information is available. See DOI: https://doi.org/10.1039/d5lp00335k.
