# Supplementary material for: Peptide-reinforced, photocrosslinkable PEG-based hydrogels
Source: RSC Appl Polym. 2026 Jan 5;4(2):704–15. doi: 10.1039/d5lp00335k (PMC12837397; doi:10.1039/d5lp00335k)
Supplement: LP-004-D5LP00335K-s001 [file LP-004-D5LP00335K-s001.pdf]

Supplementary Information (SI) for RSC Applied Polymers.
This journal is © The Royal Society of Chemistry 2025

Supplementary Information

Peptide-Reinforced Photocrosslinkable PEG-based Hydrogels

*Sam Russell, Daseul Jang, Jessica Thomas, Patrick Grysan, Linus Sprandl, Markus Biesalski, LaShanda T.J Korley, Nico Bruns**

Sam Russell, Linus Sprandl, Markus Biesalski, Nico Bruns

Department of Chemistry, Technical University of Darmstadt, Peter-Grünberg-Straße 4, 64287 Darmstadt, Germany.

Sam Russell, Nico Bruns

Centre for Synthetic Biology, Technical University of Darmstadt, Peter-Grünberg-Straße 4, 64287 Darmstadt, Germany.

Department of Pure and Applied Chemistry, University of Strathclyde, Thomas Graham Building, 295 Cathedral Street, Glasgow G1 1XL, U.K.

Patrick Grysan

Materials Research and Technology, Luxembourg Institute of Science and Technology,
5 Avenue des Hauts-Fourneaux, Esch-sur-Alzette, L-4362 Luxembourg

Daseul Jang, Jessica Thomas, LaShanda T.J Korley

Department of Materials Science and Engineering, University of Delaware, 127 The Green, 209 DuPont Hall, Newark, DE, 19716 USA

Department of Chemical and Biomolecular Engineering, University of Delaware, 150 Academy Street, Newark, DE, 19716 USA

E-Mail: nico.bruns@tu-darmstadt.de

Table S1. Summary of the weight ratios of the constituents of the polymer networks

Sample	Composition of polymer networks				Initial Reaction Mixture			
	PHEA : Crosslinker	PHEA (wt%)	PEG (wt%)	PBLA (wt%)	2-HEA (μL)	Crosslinker (mg)	Initiator (mg)	DMSO (μL)
PHEA-*l*-(PBLA$_{20}$-*b*-PEG-*b*-PBLA$_{20}$)	30:70	30	16	54	120	280		680
	50:50	50	12	38	200	200		600
	70:30	70	7	23	280	120		520
PHEA-*l*-(PBLA$_{25}$-*b*-PEG-*b*-PBLA$_{25}$)	30:70	30	20	50	120	280		680
	50:50	50	14	36	200	200	8	600
	70:30	70	9	21	280	120		520
PHEA-*l*-PEG	30:70	30	70	-	120	280		680
	50:50	50	50	-	200	200		600
	70:30	70	30	-	280	120		520

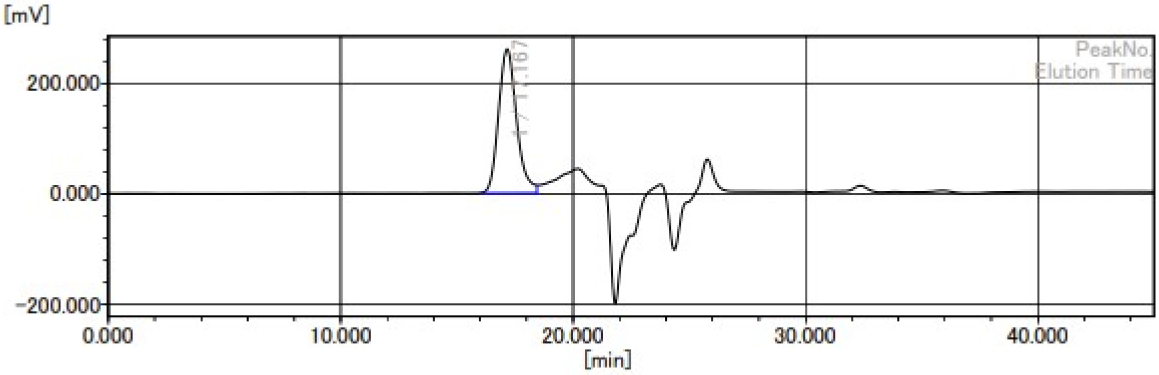

Figure S1. GPC elugram of PBLA$_{20}$-*b*-PEG-*b*-PBLA$_{20}$ triblock copolymer

	[min]	[mV]	[mol]
Peak Start	16.083	1.675	43958
Peak Top	17.167	261.567	12116
Peak End	18.450	15.083	3028
Height[mV]			260.773
Area[mV s]			14051.204
Area%[%]			100.000
[Eta]			12501.78665

Mn	10396
Mw	12502
Mz	14702
Mz+1	17029
Mv	12502
Mp	12117
Mz/Mw	1.176
Mw/Mn	1.203
Mz+1/Mw	1.362

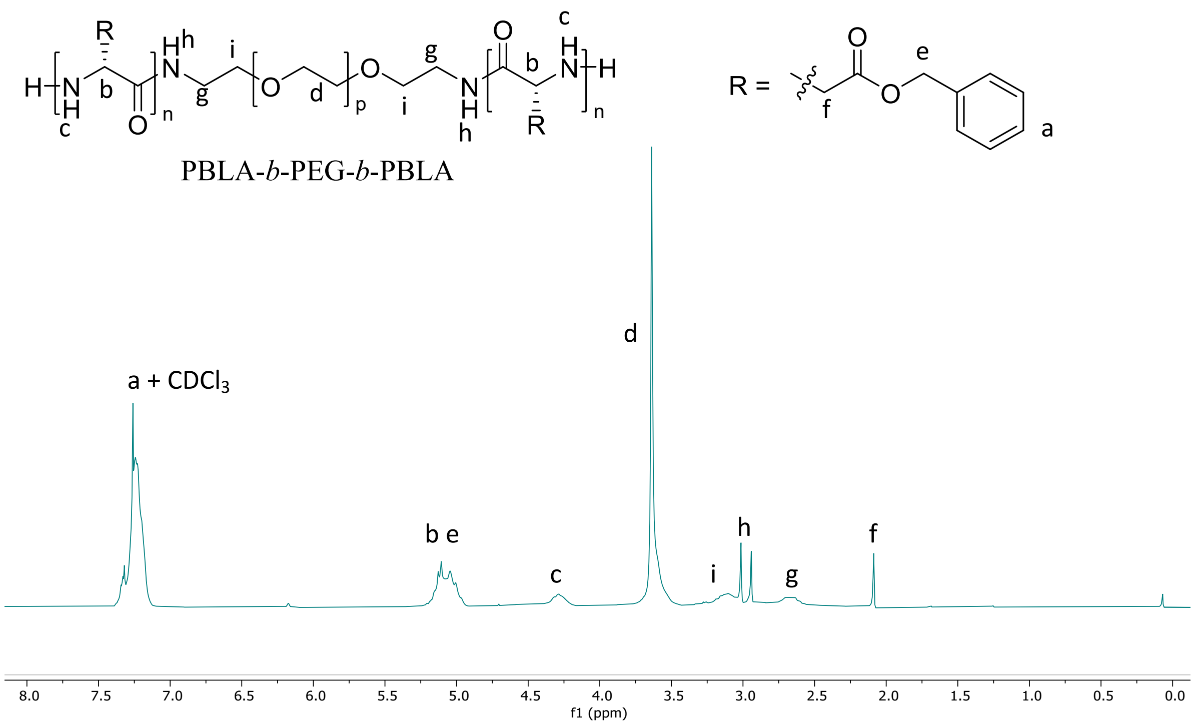

Figure S2. ^{1}H NMR spectra of PBLA-*b*-PEG-*b*-PBLA triblock copolymer

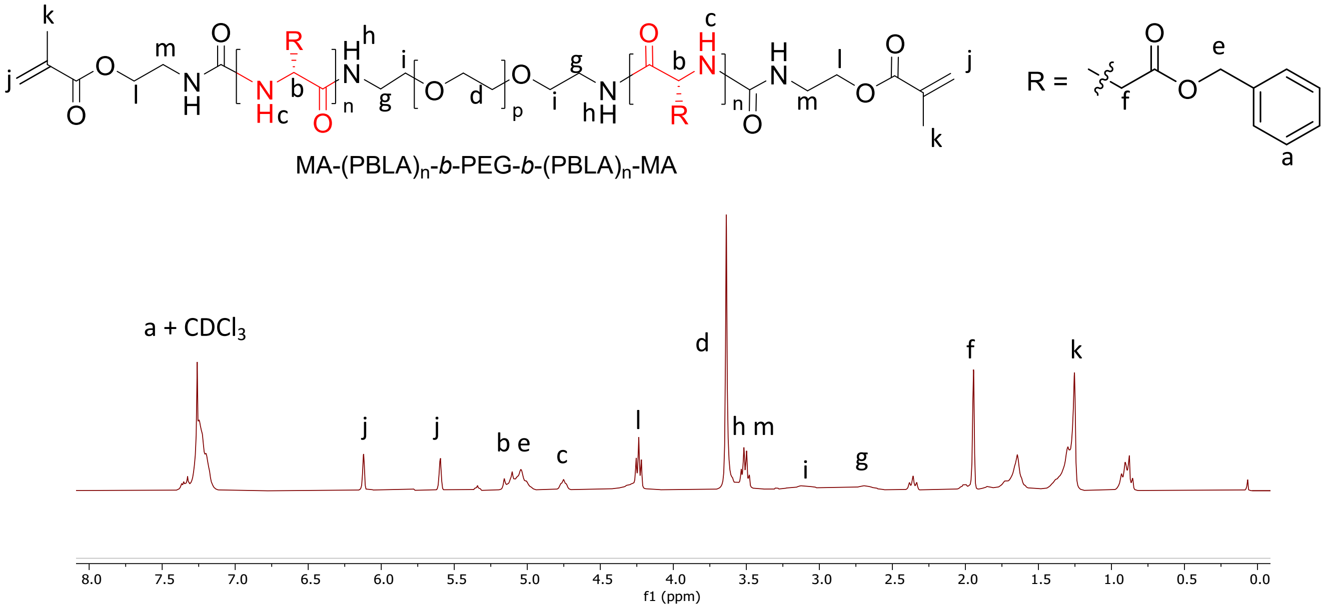

Figure S3. ^{1}H NMR spectra of MA-PBLA-*b*-PEG-*b*-PBLA-MA triblock copolymer

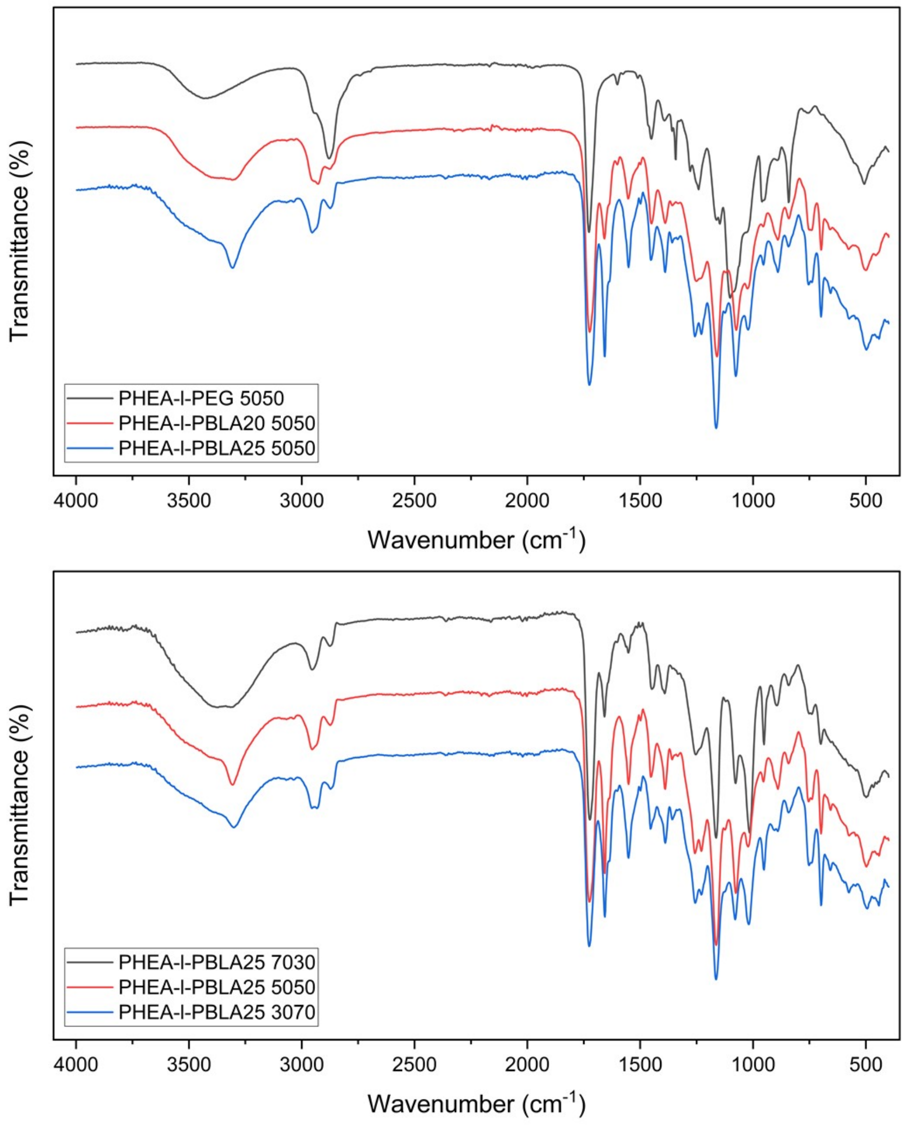

Figure S4. ATR-FT-IR spectra showing A) PHEA-*l*-PBLA$_{20}$-*b*-PEG-*b*-PBLA$_{20}$, PHEA-l-PBLA$_{25}$-*b*-PEG-*b*-PBLA$_{25}$ and PHEA-*l*-PEG, with 50:50 ratio of peptide to PHEA. B) Comparison of different compositions of PHEA-*l*-PBLA$_{25}$-*b*-PEG-*b*-PBLA$_{25}$ networks (30:70, 50:50 and 70:30 wt%).

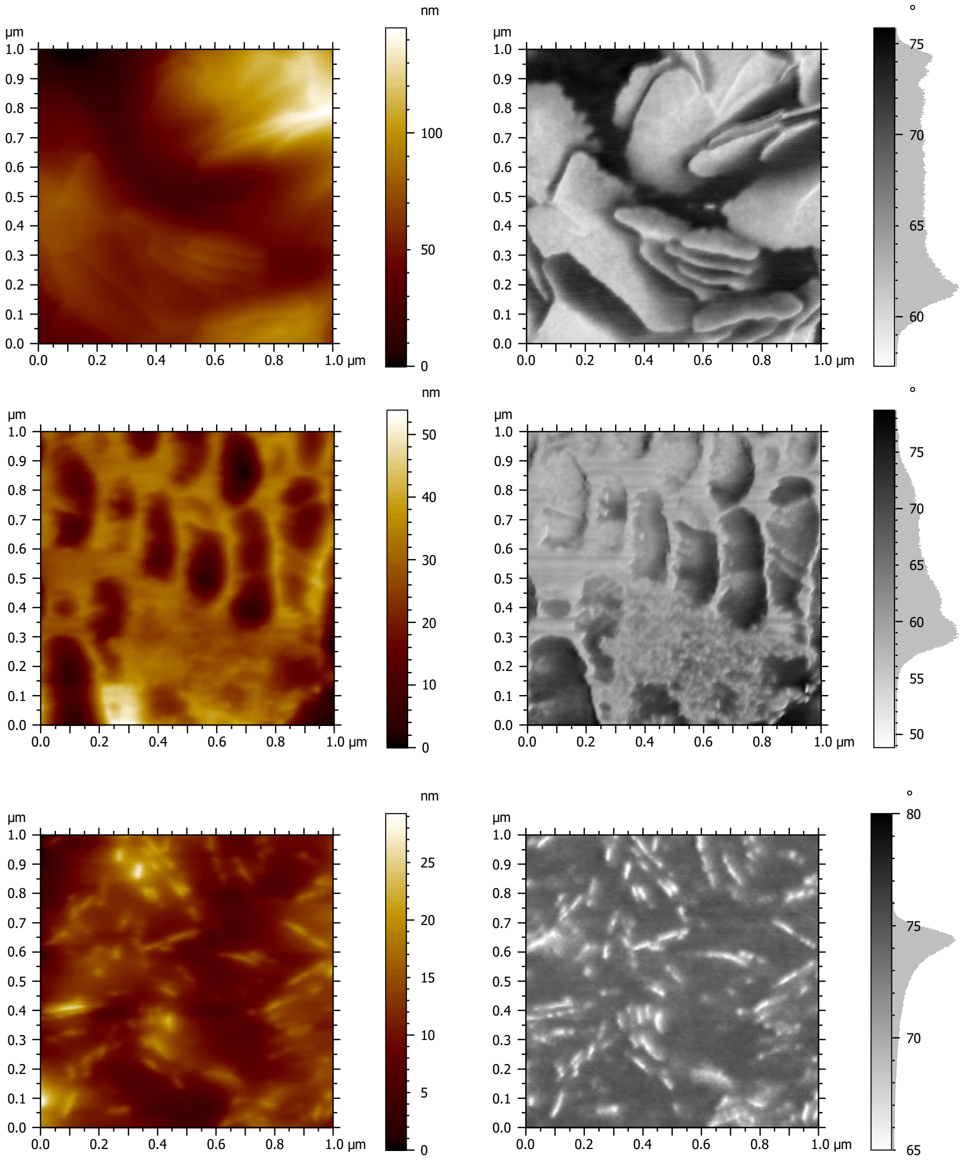

Figure S5. AFM images of PHEA-*l*-PEG 70:30 wt% networks.

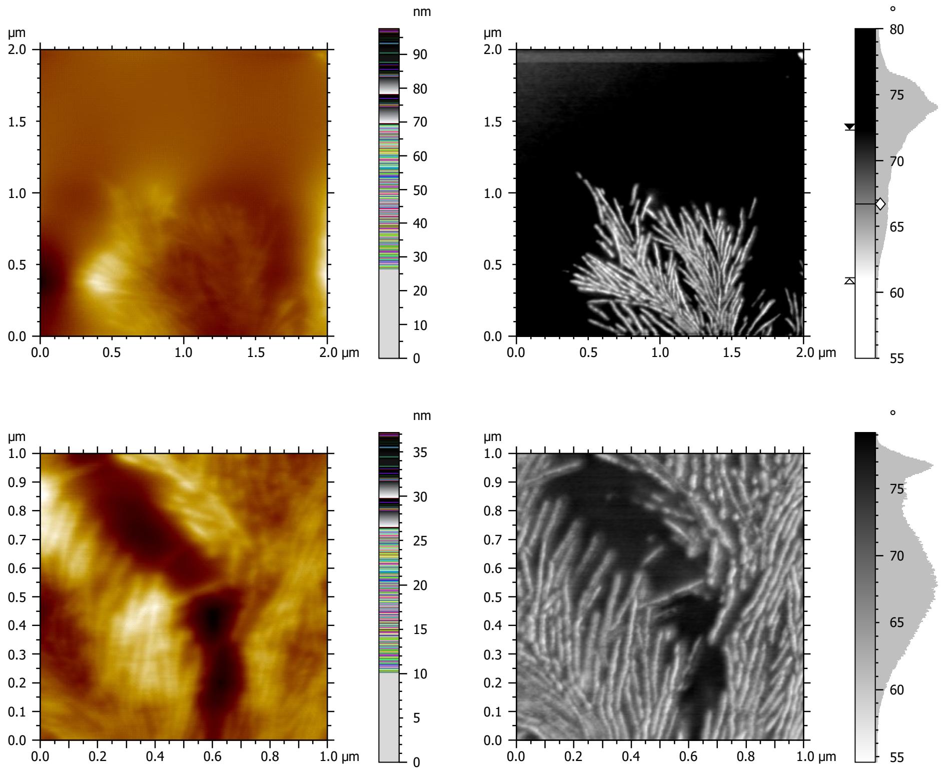

Figure S6. AFM images of PHEA-*l*-PEG 30:70 wt% networks.

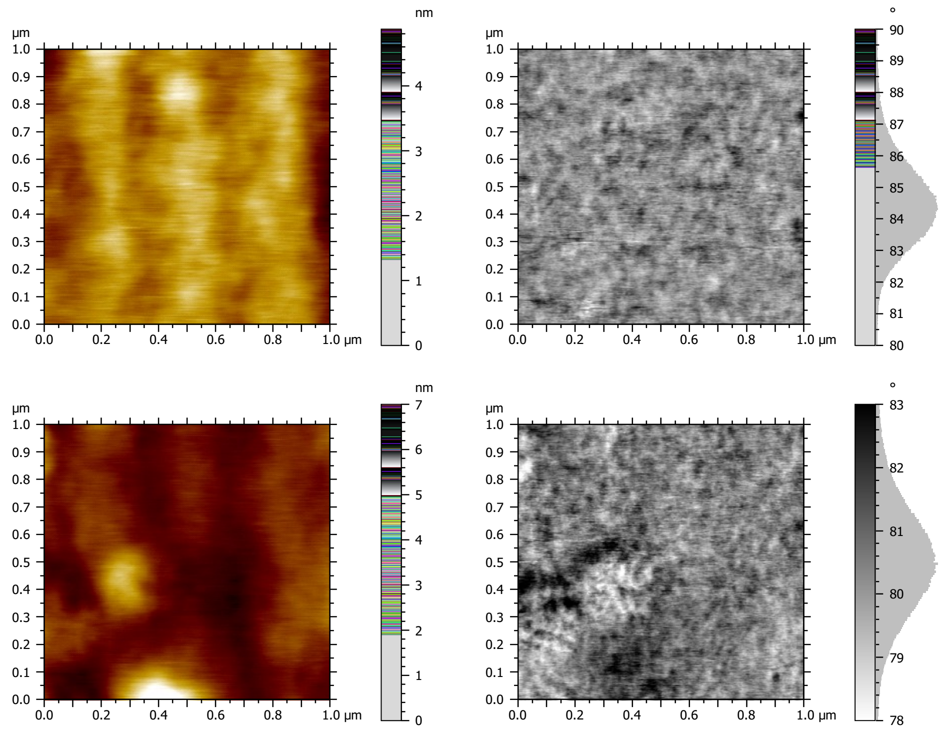

Figure S7. AFM images of PHEA-*l*-$PBLA_{20}$ 70:30 wt% networks.

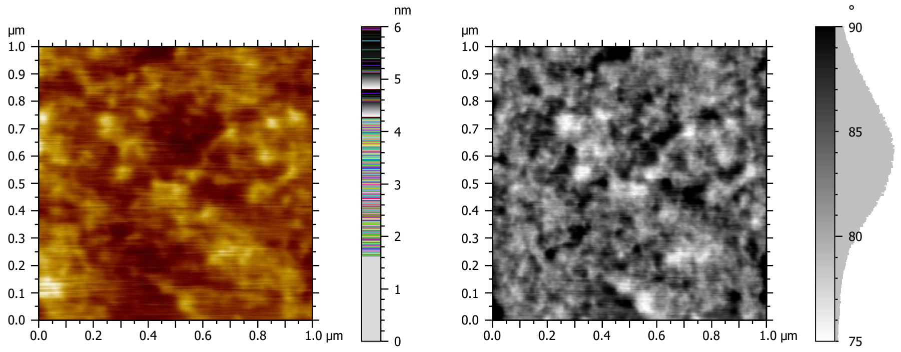

Figure S8. AFM images of PHEA-*l*-$PBLA_{20}$ 30:70 wt%.

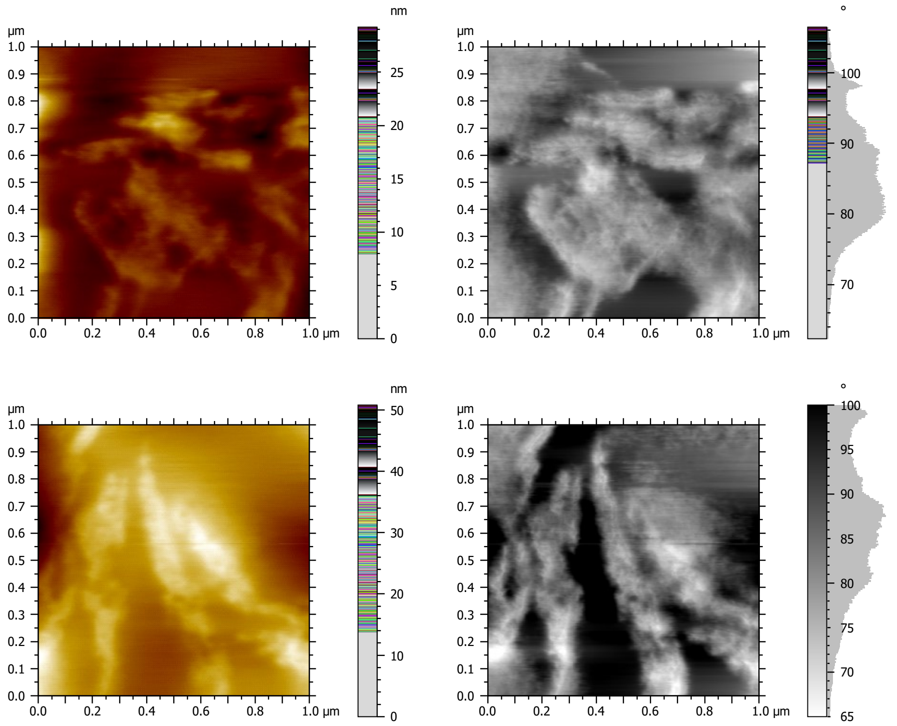

Figure S9. AFM images of PHEA-*l*-$PBLA_{25}$ 70:30 wt% networks.

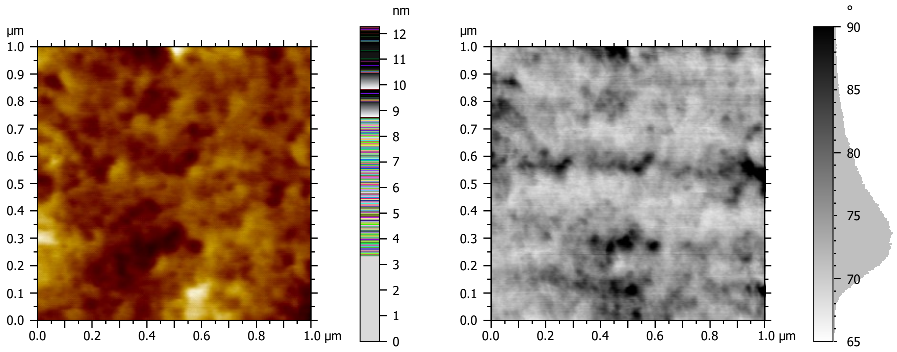

Figure S10. AFM images of PHEA-*l*-$PBLA_{25}$ 30:70 wt% networks

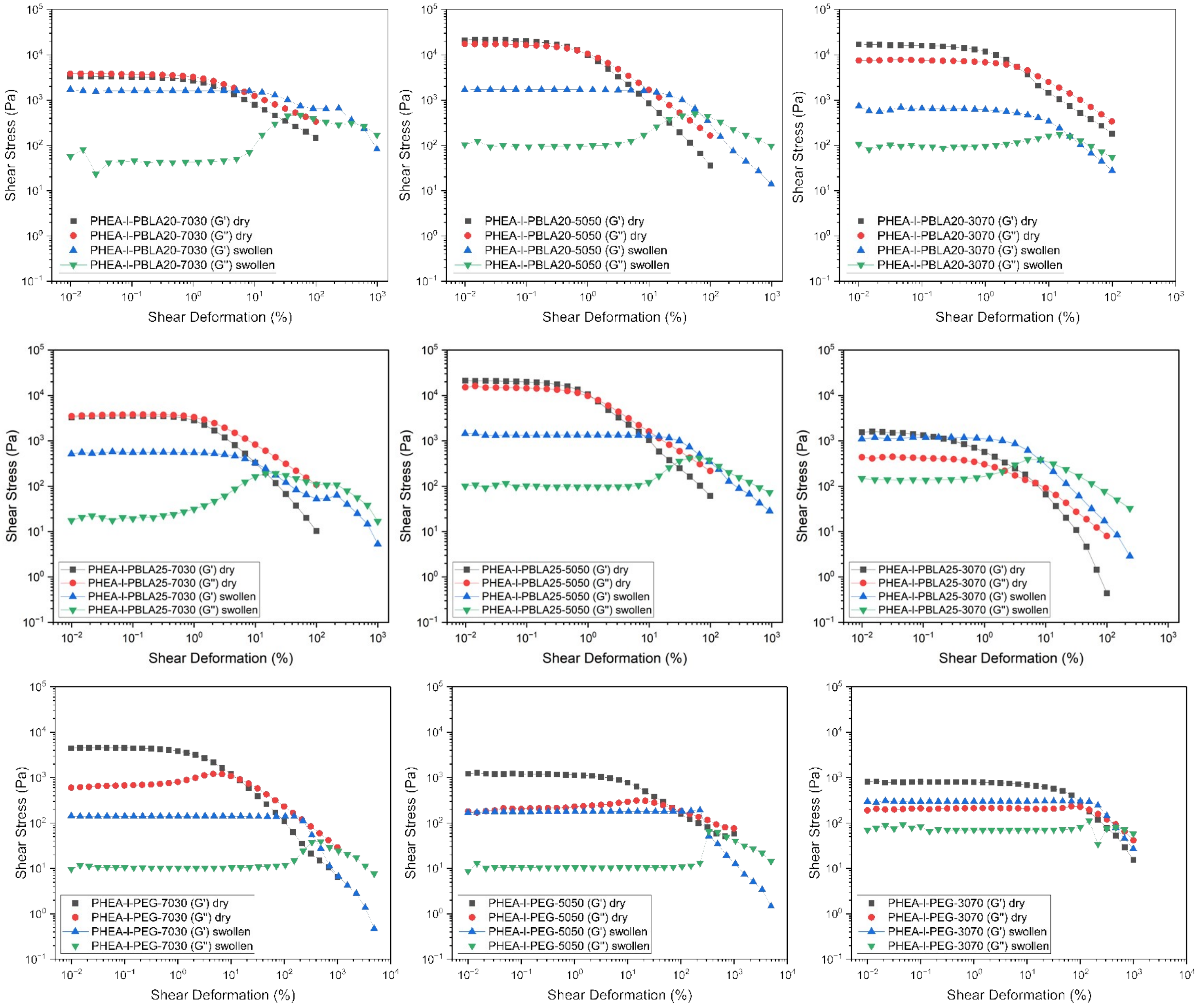

Figure S11. Rheology data of PHEA-*l*-PBLA$_{20}$, PHEA-*l*-PBLA$_{25}$ and PHEA-*l*-PEG networks in the dry and swollen state.
